# Two new species of *Coleoxestia* Aurivillius, 1912 from Brazil (Coleoptera, Cerambycidae, Cerambycinae, Cerambycini)

**DOI:** 10.3897/zookeys.612.7851

**Published:** 2016-08-23

**Authors:** Maria Helena M. Galileo, Antonio Santos-Silva

**Affiliations:** 1 PPG Biologia Animal, Departamento de Zoologia, Universidade Federal do Rio Grande do Sul, Porto Alegre, RS, Brazil. (Fellow of the Conselho Nacional de Desenvolvimento Científico e Tecnológico); 2 Museu de Zoologia, Universidade de São Paulo, São Paulo, SP, Brazil

**Keywords:** Amazonian region, Central Brazil, Neotropical Region, Sphallotrichina, taxonomy

## Abstract

Two new species of *Coleoxestia* Aurivillius, 1912 are described from Brazil: *Coleoxestia apeara*, and *Coleoxestia moromokoi*. Both are included in a previous key.

## Introduction

Currently *Coleoxestia* Aurivillius, 1912 contains 45 species distributed from North (Mexico) to South America (Monné 2015). Eya and Chemsak (2005a, 2005b) revised the species from Mexico and Central America, recognizing 12 species in the region. Martins and Monné (2005) revised the species from South America, recording 35 species in the continent.

## Material and methods

Photographs were taken with a Canon EOS Rebel T3i DSLR camera and Canon MP-E 65mm f/2.8 1–5× macro lens and focus stacking system by Zerene Stacker AutoMontage software. Measurements were taken in ‘‘mm’’ using a micrometer ocular Hensoldt/Wetzlar - Mess 10 in the Leica MZ6 stereomicroscope, also used in the study of the specimen.

The collection acronym used in this study is as follows:

INPA
 Coleção Sistemática de Entomologia, Instituto Nacional de Pesquisas da Amazônia, Manaus, Amazonas, Brazil 


## Systematics

### Cerambycini Latreille, 1802 Sphallotrichina Marins & Monné, 2002

#### Coleoxestia apeara sp. n.

Taxon classificationAnimaliaColeopteraCerambycidae

http://zoobank.org/FA534F2D-C744-4460-8A5E-97DEE28B3FD8

Figs 1 , 2 , 3 , 4

##### Description.

Holotype male. Integument dark brown, almost black; mouthparts brown, with yellowish areas; sensorial area of antennomeres dark reddish brown; femora mostly reddish brown, with base and apex dark brown; ventrites gradually lighter from I to V.

**Head.** Frons coarsely, confluently punctate laterally; diamond-shaped, tumid area close to clypeus coarsely, sparsely punctate; with short, sparse, white setae (sparser on diamond-shaped area) interspersed with some long setae close to antennal tubercle. Area between antennal tubercles deeply sulcate. Area between upper eye lobes and middle of vertex coarsely, confluently punctate; area of vertex close to margin of prothorax coarsely, abundantly punctate (punctures smaller than area closer to eyes); with very short and sparse setae, except for long, yellowish, erect setae close to upper eye lobes. Longitudinal sulcus distinct from clypeus to area between antennal tubercles. Area behind upper eye lobes coarsely confluently punctate toward eye, somewhat finer toward prothorax; with short, sparse setae. Area behind lower eye lobes coarsely, abundantly punctate close to prothorax, smooth on entire superior area close to eye, coarsely, confluently, shallowly punctate on narrow band close to inferior area of eye, smooth between this band and punctate area close to prothorax. Antennal tubercles coarsely, moderately abundantly punctate toward antennal socket, sparser toward sulcus between tubercles. Genae finely, confluently punctate, with short, sparse setae (denser close to eye), except for narrow, smooth, glabrous area close to apex; apex projected forward, subacute. Gula finely, transversely sulcate. Submentum opaque, microsculptured, coarsely, shallowly, sparsely punctate (punctures laterally confluent); with short, yellowish, moderately abundant setae interspersed with long setae (more abundant laterally). Postclypeus finely, abundantly punctate centrally, smooth laterally; with short, sparse setae on punctate area, with one long seta each side. Basal half of labrum coplanar with clypeus, finely, sparsely punctate, with short sparse setae, with tuft of very long setae laterally; distal half distinctly inclined, finely, abundantly punctate, with long, abundant setae. Distance between upper eye lobes 0.35 times length of scape; distance between lower eye lobes in frontal view 0.85 times length of scape. Antennae as long as 1.15 times elytral length; reaching elytral apex at base of antennomere XI. Scape slightly, gradually enlarged toward apex; finely, abundantly punctate; with short, sparse setae interspersed with some long setae. Antennomere III distinctly enlarged from base to apex, flattened from inner to outer surface; sensorial area wide, very distinct dorsally and ventrally, carinate on distal outer half; inner surface shiny; outer apex rounded. Antennomeres IV–X parallel-sided, with sensorial area wide and very distinct (gradually wider toward X), carinate on outer surface from base to apex; inner surface gradually more microsculptured from IV to X; distinctly flattened from inner to outer surface. Antennomere XI narrowed on distal third; entirely microsculptured. Antennal formula (ratio) based on antennomere III: scape = 0.61; pedicel = 0.15; IV = 0.77; V = 0.86; VI = 0.86; VII = 0.82; VIII = 0.78; IX = 0.78; X = 0.77; XI = 1.15.

**Thorax.** Prothorax slightly wider than long (largest width 1.1 times length); laterally with three gibbosities (central ones largest). Pronotum coarsely, abundantly punctate (punctures confluent on some areas), except for smooth central, longitudinal callosity on basal half, and subsmooth area close to anterior margin; with very short and sparse setae; with some long setae laterally on basal half. Sides of prothorax coarsely, densely, confluently punctate, except for anterior area with punctures smoother and less distinct; with very short, sparse setae interspersed with some long setae. Basal half of prosternum smooth, glabrous centrally, coarsely, abundantly punctate, with short, sparse setae laterally; anterior half transversely sulcate, coarsely, sparsely punctate on its basal third, smooth toward anterior margin, with short, sparse setae. Prosternal process smooth, glabrous centrally, with very short, abundant setae laterally; horizontal apex cordiform. Mesosternum microsculptured, coarsely, sparsely punctate; with short, abundant pubescence (not obscuring integument). Mesepimera and mesepisterna with short pubescence, not obscuring integument. Mesosternal process without tubercle, pubescent. Metepisterna with short pubescence, not obscuring integument. Metasternum finely, moderately sparsely punctate throughout, interspersed with coarse, sparse punctures each side of longitudinal sulcus; with short, abundant setae laterally, gradually sparser toward center, interspersed with long, sparse setae (more abundant each side of longitudinal sulcus). Scutellum with short, moderately sparse setae. **Elytra.** Shiny, finely, abundantly punctate, gradually finer toward apex; nearly all punctures with microscopic seta; apex somewhat obliquely truncate; outer angle with long, curved spine (outer side accompanying elytral curvature); sutural angle with distinct spine (shorter than that on outer angle). **Legs.** Apices of meso- and metafemora with rounded lobe.

**Abdomen.** Ventrites finely, sparsely punctate (slightly denser on V); ventrites I–IV laterally pubescent, with short sparse setae on remaining surface, interspersed with long setae centrally; ventrite V with short and long setae more abundant than on remaining ventrites; apex of ventrite V widely truncate.

**Dimensions in mm (male).** Total length, 23.5; length of prothorax at center, 3.8; largest width of prothorax, 4.2; anterior width of prothorax, 3.2; posterior width of prothorax, 3.6; humeral width, 5.5; elytral length, 16.8.

**Figures 1–8.**
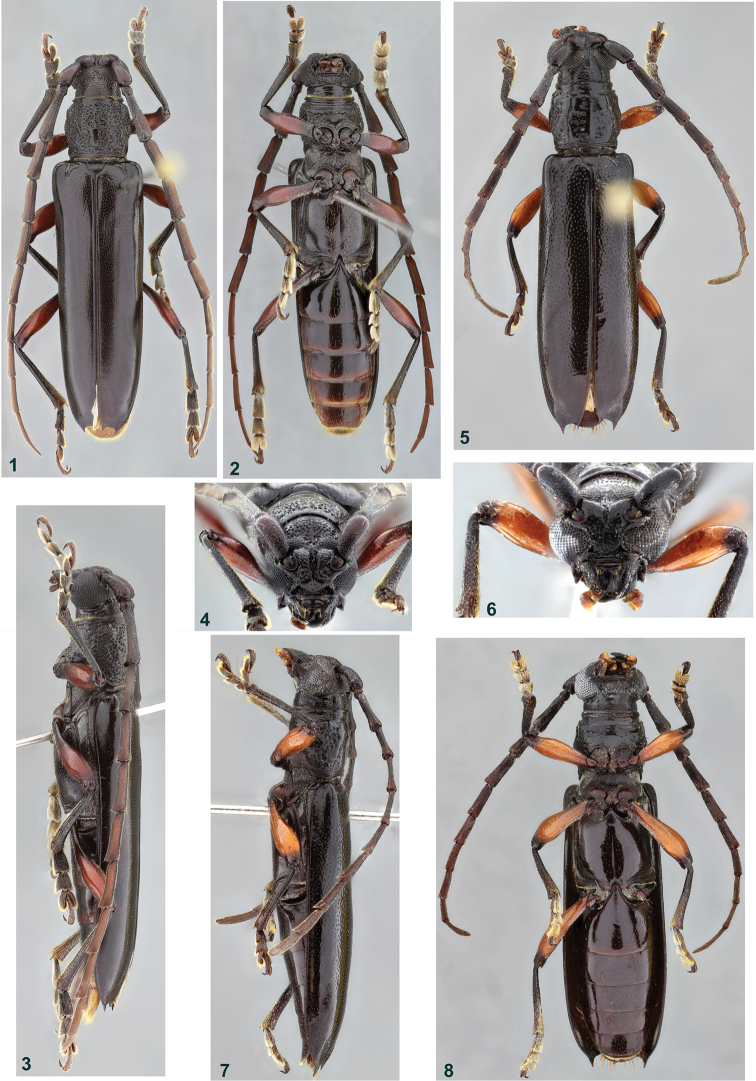
**1–4** *Coleoxestia apeara*, holotype male: **1** dorsal view **2** ventral view **3** lateral view **4** head, frontal view **5–8** *Coleoxestia moromokoi*, holotype male: **5** dorsal view **6** head, frontal view **7** lateral view **8** ventral view.

##### Type material.

Holotype male from BRAZIL, *Distrito Federal*: Brasília (Fazenda Água Limpa, Universidade de Brasília, 1050 m, 15°56'49"S / 47°56'15"W), 25.III.2008, J. A. Rafael & F. F. Xavier Filho col. (INPA).

##### Etymology.

Tupi, apéara = surface. It refers to the surface of the pronotum coarsely punctate, without striae.

##### Remarks.

*Coleoxestia apeara* sp. n. belongs to the group of species without striae on pronotum and with antennomeres distinctly explanate laterally. Only *Coleoxestia cinnamomea* (Gounelle, 1909), *Coleoxestia exotica* Martins & Monné, 2005, and *Coleoxestia ebenina* Melzer, 1935 share both features with the new species. It differs from *Coleoxestia cinnamomea* as follows: integument dark brown, almost black; elytral punctures finer; antennae in male surpassing elytral apex by antennomere XI. In *Coleoxestia cinnamomea* the integument is from orangish to reddish-brown, the elytral punctures are fine, but distinctly coarser than in the new species, and the antennae in male surpass elytral apex by antennomeres X and XI. It can be separated from *Coleoxestia exotica* by the elytra shiny (opaque in *Coleoxestia exotica*) and by the basal antennomeres distinctly wider (narrower in *Coleoxestia exotica*). It differs from *Coleoxestia ebenina* mainly by the elytra shiny (more opaque in *Coleoxestia ebenina*) and distinctly punctate (without distinct punctures in *Coleoxestia ebenina*).

*Coleoxestia apeara* sp. n. can be included in the alternative of couplet “19” from Martins and Monné (2005) (translated; modified):

**Table:** 

19(18)	Elytra shiny, not shagreened	**18**’
–	Elytra more opaque, shagreened	**20**
18’(19)	Antennomere III filiform; pronotum distinctly transversely rugose. *Coleoxestia spinipennis* (Audinet-Serville, 1834)	**36**
–	Antennomere III notably enlarged toward apex; pronotum without transverse wrinkles. Brazil (Distrito Federal)	***Coleoxestia apeara* sp. n.**

#### Coleoxestia moromokoi sp. n.

Taxon classificationAnimaliaColeopteraCerambycidae

http://zoobank.org/14318E30-C234-4C2B-A9A6-DF13749A852D

Figs 5 , 6 , 7 , 8

##### Description.

Holotype male. Integument black; mouthparts brown, with yellowish areas; antennae gradually dark-brown toward distal antennomeres; coxae brown; trochanteres and base of femora reddish-brown; remaining surface of femora orangish with black apex.

**Head.** Frons finely, moderately abundantly punctate on slightly tumid area close to clypeus, denser toward base of antennal tubercles; with deep pit each side close to clypeus; sides with carina between tumid area and antennal tubercles; with very short and sparse setae. Area between antennal tubercles sulcate. Area between upper eye lobes finely, abundantly punctate laterally, almost smooth centrally, mainly toward vertex; with moderately short, erect setae close to eyes. Vertex moderately coarsely, abundantly punctate; with very sparse, minute setae. Longitudinal sulcus slightly distinct from clypeus to antennal tubercles, becoming carina-shaped up to middle of upper eye lobes. Area behind upper eye lobes with sculpture as on vertex; with moderately short setae close to eyes and minute, very sparse setae on remaining surface. Area behind lower eye lobes longitudinally, widely sulcate close to eyes (almost smooth on this region), moderately coarsely, abundantly punctate toward prothorax. Antennal tubercles finely, sparsely punctate; with minute, sparse setae. Genae finely, moderately abundantly punctate; with minute, sparse setae. Gula shiny, smooth. Submentum microsculptured; with transverse row of coarse punctures close to gula; with short, moderately abundant setae interspersed with some long setae. Postclypeus moderately coarsely, confluently punctate centrally, smooth laterally; with very sparse, minute setae. Basal half of labrum coplanar with clypeus, and distal half distinctly inclined; coplanar region with long setae laterally. Distance between upper eye lobes 0.3 times length of scape; distance between lower eye lobes in frontal view 0.6 times length of scape. Antennae as long as 1.3 times elytral length; almost reaching elytral apex. Scape almost parallel-sided; finely, moderately abundantly punctate (mainly on basal third); with minute, sparse setae. Outer apex of antennomeres III–IV nodose; outer apex of antennomeres V–X dentate. Antennal formula (ratio) based on antennomere III: scape = 0.85; pedicel = 0.20; IV = 0.88; V = 0.94; VI = 0.88; VII = 0.88; VIII = 0.82; IX = 0.85; X = 0.82; XI = 1.23.

**Thorax.** Prothorax slightly wider than long (largest width 1.1 times length); laterally with three, slightly distinct gibbosities (central ones more distinct). Pronotum coarsely punctate on wide, longitudinal band each side of smooth central region, interspersed with fine, sparse punctures (area with coarse punctures slender middle, not reaching anterior margin); coarsely, confluently punctate laterally at basal 4/5; between these two coarsely punctate area with wide band with sparse, coarse and fine punctures; anterior fifth with transverse, well-marked sulcus and fine, sparse punctures; with minute and sparse setae (slightly more distinct laterally). Sides of prothorax coarsely, moderately abundantly punctate on wide central region (punctures confluent on some regions); with minute and sparse setae; with some long setae centrally close to pronotum; basal area smooth; anterior fifth with irregular sculpture. Prosternum finely, sparsely punctate on basal half (sparser centrally), with very sparse, minute setae; anterior half with two transverse sulci (the most distal distinctly narrow). Prosternal process glabrous centrally, with short, abundant setae laterally; horizontal apex somewhat rounded. Mesosternum with short, abundant setae (not obscuring integument). Mesepimera and mesepisterna with pubescence slightly denser than on mesosternum. Mesosternal process without tubercle, pubescent; apex deeply emarginate centrally. Metepisterna with short pubescence, not obscuring integument. Metasternum finely, sparsely punctate throughout; with short, moderately abundant setae on narrow lateral band and transverse area close to metacoxae; remaining surface with short, sparse setae interspersed with some long setae. Scutellum finely, abundantly punctate laterally, smooth centrally; with short setae laterally. **Elytra.** Shiny, finely, abundantly punctate throughout (punctures finer toward apex); apex somewhat obliquely truncate; outer angle with long spine; sutural angle with spine very distinct, but shorter than that of outer angle. **Legs.** Apices of meso- and metafemora with rounded lobe.

**Abdomen.** Ventrites finely, sparsely punctate; with short, sparse setae interspersed with some long setae; apex of ventrite V subrounded, slightly emarginate centrally.

**Dimensions in mm (male).** Total length, 15.10; length of prothorax at center, 2.40; largest width of prothorax, 2.75; anterior width of prothorax, 2.15; posterior width of prothorax, 2.30; humeral width, 3.30; elytral length, 10.4.

##### Type material.

Holotype male from BRAZIL, *Amazonas*: Ipixuna (Rio Liberdade, Estirão da Preta, 07°21'46.7"S / 71°52'07.1"W), 11-15.V.2011, J. A. Rafael, J. T. Câmara, R. F. Silva, A. Somavilla, and C. Gonçalves col. (INPA).

##### Etymology.

Tupi, moro = color; mokõî = two. Allusive to the femora orange with black apex.

##### Remarks.

*Coleoxestia moromokoi* sp. n. belongs to the group of species with femora reddish or orangish, distinctly contrasting with dark apex, and with elytral apex bispinose: *Coleoxestia anthracina* Martins & Monné, 2005; *Coleoxestia brevipennis* (Bates, 1870); *Coleoxestia femorata* (Gounelle, 1909); *Coleoxestia guttula* Martins & Monné, 2005; *Coleoxestia polita* (Waterhouse, 1870); *Coleoxestia sanguinipes* (Bates, 1884); *Coleoxestia tupunhuma* Martins & Monné, 2005. *Coleoxestia moromokoi* sp. n. differs from all these species by the pronotum not transversely rugose, while in these other species it is distinctly rugose.

*Coleoxestia moromokoi* sp. n. can be included in the alternative couplets “14” from Martins and Monné (2005) (translated):

**Table:** 

14’(13)	Pronotum not transversely rugose. Brazil (Amazonas)	***Coleoxestia moromokoi* sp. n.**
–	Pronotum transversely rugose	**14**

## Supplementary Material

XML Treatment for Coleoxestia apeara

XML Treatment for Coleoxestia moromokoi
